# Get ready for short tandem repeats analysis using long reads-the challenges and the state of the art

**DOI:** 10.3389/fgene.2025.1610026

**Published:** 2025-07-02

**Authors:** Marija Chaushevska, Karmele Alapont-Celaya, Anne Kristine Schack, Lukasz Krych, M. Carmen Garrido Navas, Anastasia Krithara, Gjorgji Madjarov

**Affiliations:** ^1^ Faculty of Computer Science and Engineering, University Saints Cyril and Methodius, Skopje, North Macedonia; ^2^ gMendel ApS, Copenhagen, Denmark; ^3^ Food Microbiology and Fermentation, Department of Food Science, University of Copenhagen, Copenhagen, Denmark; ^4^ Cardiovascular Development Group, Department of Experimental Biology, University of Jaen, Jaen, Spain; ^5^ GENetic COUNselling (CONGEN), Genetic Counselling Services, Granada, Spain; ^6^ Institute of Informatics and Telecommunications, National Centre of Scientific Research “Demokritos”, Athens, Greece

**Keywords:** short tandem repeats, long reads, sequencing technologies, structural variants, variant detection, bioinformatics tools

## Abstract

Short tandem repeats (STRs) are repetitive DNA sequences that contribute to genetic diversity and play a significant role in disease susceptibility. The human genome contains approximately 1.5 million STR loci, collectively covering around 3% of the total sequence. Certain repeat expansions can significantly impact cellular function by altering protein synthesis, impairing DNA repair, and leading to neurodegenerative and neuromuscular diseases. Traditional short-read sequencing struggles to accurately characterize STRs due to its limited read length, which limits the ability to resolve repeat expansions, increases mapping errors, and reduces sensitivity for detecting large insertions or interruptions. This review examines how long-read sequencing technologies, particularly Oxford Nanopore and PacBio, overcome these limitations by enabling direct sequencing of full STR regions with improved accuracy. We discuss challenges in sequencing, bioinformatics workflows, and the latest computational tools for STR detection. Additionally, we highlight the strengths and limitations of different methods, providing deeper insight into the future of STR genotyping.

## 1 Introduction

Over the years, various advances have been made in the detection of genetic variations and mutations within DNA. Primary mutations in DNA encompass various types, each with distinct implications for genetic diversity and disease susceptibility. Genetic variants can be broadly categorized into different types, each representing specific alterations in the DNA sequence. Single Nucleotide Polymorphisms (SNPs) Rafalski (2002) represent the most prevalent form of genetic variation, where a single nucleotide position can differ between individuals, contributing to both normal genetic diversity and disease susceptibility. Point mutations, often used interchangeably with SNPs, involve the substitution of a single nucleotide base, which can lead to changes in amino acid sequences and potentially affect protein function. Indels Mullaney et al. (2010), short for insertions and deletions, are small variants that involve the addition or removal of nucleotides, typically less than 49 bp; larger insertions and deletions fall into the category of structural variants (SVs). Copy number variations (CNVs) Zhao et al. (2013) involve changes in the number of copies of a particular DNA segment, which influence gene dosage and can contribute to diseases. They are also considered a subcategory of SVs, as they affect the genomic structure and gene dosage. Structural variants (SVs) Feuk et al. (2006); Byrska-Bishop et al. (2022) encompass larger-scale alterations, including inversions, translocations, and large insertions or deletions, impacting the overall architecture of the genome Sudmant et al. (2015). Commonly found in the human genome, these variations arise from crucial biological processes such as DNA replication, repair, meiotic recombination, and retrotransposition, in addition to single nucleotide variations (SNVs) and small insertions or deletions (indels) Bickhart and Liu (2014). In contrast to the most prevalent SNVs, structural variants (SVs) contribute 3.4 times more nucleotides to human genetic diversity Huddleston et al. (2017).

Another important class of variations involves highly repetitive sequences known as Short Tandem Repeats (STRs). STRs, also called microsatellites, are different from traditional structural variants in that they consist of short nucleotide motifs (2–6 base pairs) repeated in tandem Tanudisastro et al. (2024); Fan and Chu (2007); Richard et al. (2008). Unlike SNPs or small indels, which typically alter single nucleotides or small stretches of DNA, STRs exhibit a unique form of genetic variability—repeat expansion and contraction. This dynamic nature makes STRs highly polymorphic and particularly relevant in forensic genetics, population studies, and various hereditary disorders. In some cases, extreme expansions of STR regions can be classified as SVs, as they can significantly alter the architecture of the genome and contribute to disease development. Given their biological significance and technical challenges in sequencing, STRs require specialized analytical approaches, which will be explored in the subsequent sections.

Our understanding of the different genetic variations and mutations has advanced significantly with the introduction of various DNA sequencing technologies that have evolved over the past few decades, becoming faster, more accurate, and more affordable. These advances are categorized into three generations, each with novel methods for decoding genetic information.

First-generation sequencing, pioneered by Sanger sequencing, laid the foundation with high accuracy but low throughput. This method relies on chain termination using dideoxynucleotides (ddNTPs) to generate DNA fragments of varying lengths, which are then separated by gel or capillary electrophoresis. Although relatively slow and labor-intensive, Sanger sequencing remains highly accurate, making it the gold standard for small-scale sequencing projects, such as single-gene analysis and validation of next-generation sequencing (NGS) results.

Second-generation sequencing or Next-generation sequencing (NGS) revolutionized genomics by introducing massively parallel sequencing, significantly reducing costs and increasing data output Hu et al. (2021). Numerous computational tools are specifically built and dedicated to short-read data mining. They are beneficial for applications requiring fast generation of a large volume of data, such as genome sequencing, transcriptomics, and metagenomics. Short reads, on the other hand, do have constraints. They struggle to resolve complicated regions of the genome, repetitive sequences, and structural changes because their short length makes it difficult to efficiently span these areas Hu et al. (2021). Detection of structural variants (SVs) from short read sequencing involves a significant false discovery rate (up to 85%) and a low sensitivity (30%–70%) Sedlazeck et al. (2018a). As a result, short-read technologies can overlook critical genetic information, compromising thorough knowledge of genomes. In addition, assembling and analyzing complex genomic regions, such as short tandem repeats, can be more time-consuming with short-read technologies due to increased computational demands compared to long-read sequencing Treangen and Salzberg (2012); Ebert et al. (2021). Larger variations in the sequence are difficult to detect with short reads, even if they work well to identify single nucleotide variations (SNVs) and small insertions and deletions (indels) Mahmoud et al. (2019); Depienne and Mandel (2021).

To overcome these challenges, Third-generation sequencing (long-read sequencing) emerged, allowing direct sequencing of much longer DNA fragments, often exceeding 10,000 base pairs Wang et al. (2021). Technologies such as PacBio SMRT and Oxford Nanopore enable real-time sequencing and better detection of structural variations, although initially with higher error rates Jeanjean et al. (2025). Both sequencing technologies have substantial base error rates (varying from 3% to 15% Luo et al. (2020)), with the majority of errors caused by insertions or deletions (indels); however, the error distribution varies Jain et al. (2018); Carneiro et al. (2012); Jain et al. (2015). Therefore, long reads can span entire SVs in many cases and achieve better mappability in repetitive genomic regions. They make it possible to identify long-range haplotypes, small indels, SVs, variations in the coding sections of genes including several pseudogenes, and phasing of distant alleles in complex genomic regions Olson et al. (2022). So, they are particularly good at resolving complex genomic regions, repeated sequences, and structural variations, giving researchers a more detailed understanding of the genome’s architecture.

Compared to short-read sequencing, long-read sequencing can identify 3 to 4 times as many SVs, particularly in the 50–1000 bp region Audano et al. (2019); Chaisson et al. (2015). Repetitive DNA sequences, which are characterized by variable tandem repeats, pose unique challenges for analysis. Long reads, capable of capturing entire repeat units in a single sequence, offer a revolutionary approach, enabling a more comprehensive understanding of the diversity, structural complexity, and potential links of STRs to genomic variability and disease. Recent studies have demonstrated this potential by profiling STR variation on a genome-wide scale using long-read sequencing technologies, offering reference resources and variability indices for diverse populations Liu et al. (2022). However, long-read sequencing comes with various drawbacks. In comparison to short-read technology, it incurs a higher cost per base pair. Moreover, error rates in long reads are often higher, complicating data processing and necessitating additional computational resources for correction Zhang H. et al. (2020).

Despite advances in sequencing technologies, accurately detecting and analyzing certain genetic variations, particularly structural variants and short tandem repeats (STRs), remains a challenge. STRs, with their highly repetitive nature and dynamic variability, pose significant obstacles in genomic analysis, especially when using traditional short-read sequencing. While long-read sequencing offers a promising approach for resolving these complex regions, it also introduces unique challenges related to error rates and bioinformatic processing.

This paper provides a comprehensive overview of the challenges and state-of-the-art methodologies and approaches associated with the genotyping workflow for STR mutations, covering the entire process from DNA extraction to variant calling. The challenges and methodologies discussed involve the use of TGS technology (long reads), because most of the structural variations, especially STRs, could be found in long reads. The primary motivation for conducting a review of STRs is their propensity to present challenges in long-read DNA sequencing. Long-read technologies can span STRs but face difficulties in accurately characterizing their complex and variable nature, and they may suffer from higher error rates and homopolymer inaccuracies. Specialized tools and algorithms have been developed to detect STRs in a human genome and mitigate their challenges. In this study, our objective is to comprehensively examine and elucidate the detection of STRs mutations within long reads. Consequently, we examine the entire workflow, beginning with library preparation, progressing through the utilization of long-read sequencing technologies for generating extended sequences, and covering the sequence alignment and variant calling processes. In addition, we address and confront challenges that may arise at each specific stage of the workflow. In addition, we review the bioinformatic tools employed at each step of the workflow to effectively address the challenges associated with STRs.

## 2 Short tandem repeats (STRs)

STRs account for about 3% of the genome and can be found in the genomes of many organisms, including humans, and certain repeat expansions could be associated with human diseases Shi et al. (2023); Hannan (2018); Subramanian et al. (2003). In addition, a large number of short tandem repeats (STRs) originate from other repeated elements, including Alu elements and short interspersed nuclear elements (SINE) and long interspersed nuclear elements (LINE) Grandi and An (2013). They are becoming increasingly popular as a tool for a variety of applications despite the fact that their mutation rates vary greatly. Even low estimations show that STRs are 3-4 orders of magnitude greater than random point mutations Ellegren (2004). From a biological point of view, due to their location in exons, introns, and intragenic regions, STRs can affect cellular function at many different levels Hannan (2010). As the first findings demonstrated, there are two primary categories of STR expansions: those that impact coding regions, mainly resulting in abnormally extended polyglutamine (polyQ, primarily encoded by CAG codons) or polyalanine (polyA, primarily encoded by GCN codons) stretches within proteins, and those that impact non-coding regions of genes Hannan (2018).

From a DNA point of view, repetitive sequences are recognized for their regulatory role in DNA transcription by activating or inactivating different genes. As an illustration, within the promoter region of the AHR gene, there exists the GGGC short tandem repeat (STR), and the expression level varies according to the number of repetitions Spink et al. (2015). The overexpression of *PCA3* has been associated with the pathogenesis of prostate cancer. It was shown that the more TAAA repeats in the *PCA3* promoter, the higher the risk of prostate cancer in a Chinese population Zhou et al. (2011). One of the mechanisms to regulate DNA expression is the formation or inhibition of binding sites to transcription factors Contente et al. (2002); Hannan (2018). STRs can also affect the formation of the secondary structure of DNA, leading to heterochromatin formation and epigenetic modifications, such as DNA methylation, which can lead to genetic silencing. This is particularly true when the repetitive fragment is located on a CpG island Hannan (2018); Wright and Todd (2023). Short CAG/CTG sequences incorporate nucleosomes and, depending on the STR length and flaking sequence, it will affect chromatin structure and transcription of nearby genes Volle and Delaney (2012). The opposite effect is observed with other STRs, such as CGG repeats Wang (2007).

At the RNA level, STRs can serve as RNA localization signals, regulate RNA translation, or affect RNA spicing, among others. STRs in 3′ untranslated regions (UTRs) sometimes serve as RNA localization signals, where they regulate the transport of RNAs to different cellular regions by interacting with different RNA binding proteins (RBPs) Wright and Todd (2023). Instead, if they are located in the 5′-UTRs, they regulate mRNA translation. GC-rich STRs can form stable RNA structures, which can impede the formation of the translation complex. When the STR size is larger, mRNA translation tends to proceed at a slower pace compared to the situation where the STR is smaller Wright and Todd (2023). When STRs are located in the introns, they can affect splicing, especially when the repeat sequence contains CA and TG dinucleotides, as they can produce new alternative splice sites Hannan (2018); Wright and Todd (2023). In addition, if STRs lead to changes in the 3D structure of the mRNA, it could cause alternative splicing by binding or inhibiting the binding of splicing factors Wright and Todd (2023).

At the protein level, when STRs are translated into amino acid sequences, they can form complex tertiary structures that can affect the function and cellular localization of the protein Wright and Todd (2023).

Besides their native functions, STRs exhibit significant polymorphisms and are linked to a wide spectrum of phenotypic variations, including some that result in neurodegenerative diseases in humans. These diseases are commonly caused by repeat expansions that affect DNA, RNA, or protein function Wright and Todd (2023); Hannan (2018). Tandem repeat disorders (TRDs) are a category of neuropathological conditions associated with the accumulation of short tandem repeats Ryan (2019). The mutation rate of TRDs is significantly impacted not only by the length of the repeat tract but also by other intrinsic qualities such as the size of the repeated unit and the purity (absence of discontinuities) of the repeated sequence. Mutations can occur during both meiosis and mitosis and lead to a high rate of somatic mutations that can affect genetic plasticity in development, biological functions, and human disease. These somatic tandem repeat mutations have been linked to several types of cancer and other TRDs Wright and Todd (2023); Salipante et al. (2014). They account for 60–70 heritable neuropathologies Chen et al. (2025); Mirkin (2007); Paulson (2018), including Huntington’s disease (CAG repeats on the short arm of chromosome 4p16 in the Huntingtin (*HTT*) gene) Walker (2007); MacDonald et al. (1993), Fragile X Syndrome (CGG repeats within the 5′ UTR in the *FMR1* gene) Saldarriaga et al. (2014); Kremer et al. (1991), Kennedy’s disease (CAG repeats on the Xq11-q12 band of the long arm of the X chromosome) Fischbeck (1997), myotonic dystrophy and several spinocerebellar ataxias Ellegren (2004). A number of TRDs, including Huntington’s disease, occur in the context of expanded glutamine (CAG) repeats, accompanied by protein misfolding, aggregation, and toxicity. The length of the repetitive region in the HTT gene (CAG repeat) in the normal population ranges from 10 to 35, while in patients with HD ranges from 36 to 121, with a reduced penetrance at repeat sizes of 36–39. Individuals with longer repeats often experience earlier onset and more severe symptoms, including motor dysfunction and cognitive decline. The repeat in the FMR1 gene is up to 55 CGGs long in the normal population. In patients with Fragile X Syndrome, a repeat length exceeding 200 CGGs (full mutation: FM) generally leads to methylation of the repeat and promoter region, which is accompanied by silencing of the FMR1 gene Willemsen et al. (2011). Weakness, atrophy, and fasciculations of the appendicular and bulbar muscles are symptoms of Kennedy’s disease, also known as X-linked spinal and bulbar muscular atrophy. The amplification of the CAG repeat of the androgen receptor gene is what causes the disease. Patients with Kennedy disease have more than 39 CAG repeats Alves et al. (2018). A Myotonic Dystrophy Thornton (2014), a multisystemic disorder, is associated with an expanded repeat of CTG in the DMPK gene. The length of the repetitive region is associated with the age of onset and severity of symptoms, which include muscle wasting, myotonia, and cardiac abnormalities. For many repeat expansion disorders, including all polyQ and many of the non-coding expansions, there are strong established correlations between the magnitude of the expansion and the age at onset and/or severity of the disorder. The phenotypic becomes more severe and the age of onset is earlier when the expansion is larger Depienne and Mandel (2021). Most STRs are found mainly in the non-coding regions of the genome, while only about 8% are located in the coding regions of the genome Gymrek (2017). Moreover, their densities vary slightly among chromosomes. In humans, chromosome 19 has the highest density of STRs. On average, one STR occurs per 2,000 bp in the human genome. The most common STRs in humans are A-rich units: A, AC, AAAN, AAN, and AG. They are generally more polymorphic than other types of variation such as sequence copy number and single-nucleotide polymorphisms Verstrepen et al. (2005).

**FIGURE 1:**
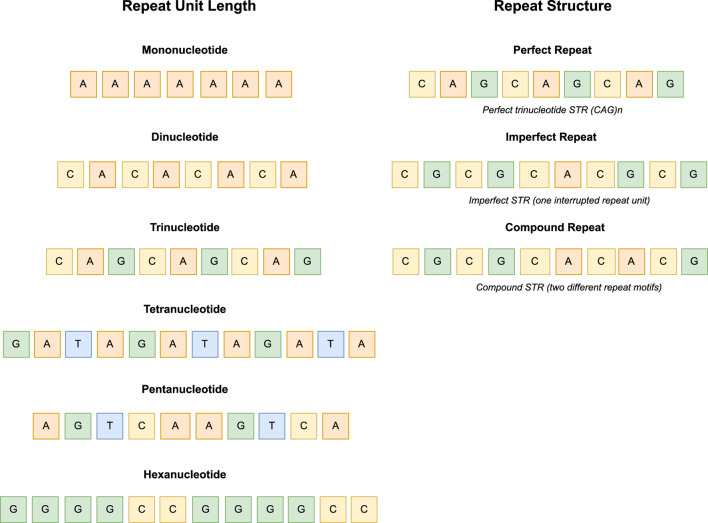
Representation of STR types based on repeat unit length (-mono, -di, -tri, -tetra, -penta and hexanucleotide) and repeat structure (perfect, imperfect and compound).

On the basis of different repeat units, STRs can be classified into different types. On the one hand, according to the length of the major repeat unit, STRs are classified into mono-, di-, tri-, tetra-, penta-, and hexanucleotide repeats. Under normal conditions, these repeat tracts are stable and short (commonly 40–70 bp), but unstable when their lengths range from 100 to 150 nucleotide bases to thousands of repeat units depending on the disease, sequence, and genomic context. Examining the variations of STRs, especially extended STRs, represents a crucial stage in understanding their variations among individuals and the processes responsible for their tendency to become unstable. The most common STRs in the human genome are dinucleotide repeats. On the other hand, according to the repeat structure, STRs are classified into perfect repeats (simple repeats), containing only one repetitive unit, imperfect repeats containing one interrupted repeat unit, and compound repeats consisting of two or more different repeat motifs arranged adjacent to each other (see Figure 1) Fan and Chu (2007), Urquhart et al. (1994).

Besides their role in medical genetics (explained earlier in this section), STRs are widely used in applications such as the construction of genetic maps Dib et al. (1996), gene localization, forensics Budowle and Sajantila (2024), genetic genealogy, genetic linkage analysis, identification of individuals, paternity testing, disease diagnosis La Spada et al. (1992), Kayser (2017), Alonso et al. (2018), population genetics Xie (2024), Fan and Chu (2007), and tracing cell lineages in cancer samples Frumkin et al. (2008). STRs are ideal markers for creating high-resolution genetic maps and for locating genes by co-segregation with phenotypic traits because of their great polymorphism and abundance over the genome. Since STRs can specifically identify individuals, even among close relatives, they form the basis of DNA profiling systems in forensic science Budowle and Sajantila (2024). Through national DNA databases, they are also frequently employed in criminal investigations and paternity testing. Y-STR haplotyping is often used in genetic genealogy to identify paternal lineages and ancestral roots. Particularly in high-density tracking of inheritance patterns, STRs remain important in linkage analysis for mapping disease-associated loci. Somatic mutations in STRs can provide molecular barcodes for lineage tracing in cancer research, thus illuminating clonal evolution and tumor heterogeneity Frumkin et al. (2008). Furthermore, their high mutation rates make STRs especially valuable in population genetics and evolutionary research, since they help to reconstruct demographic history, evaluate genetic diversity, and track conservation initiatives Xie (2024).

The main challenge when analyzing STRs is that they are a common source of systematic sequencing and mapping errors and frequently cause structural variants. The developments in sequencing technologies and bioinformatics tools in the past few years have renewed interest in the detection of STR variation from high-throughput sequencing (HTS) data. Advances in sequencing allow for the generation of longer reads, providing more information for the detection of STRs length variation Jeanjean et al. (2025). New sophisticated alignment methods that are indel (insertion or deletion) tolerant have been developed, enabling a more accurate alignment of reads in STR loci. Importantly, several tools Cao et al. (2014); Gymrek et al. (2012); Highnam et al. (2013) for STR genotyping have come out in the past years.

## 3 From DNA to variant calling

### 3.1 STRs analysis workflow

**FIGURE 2:**
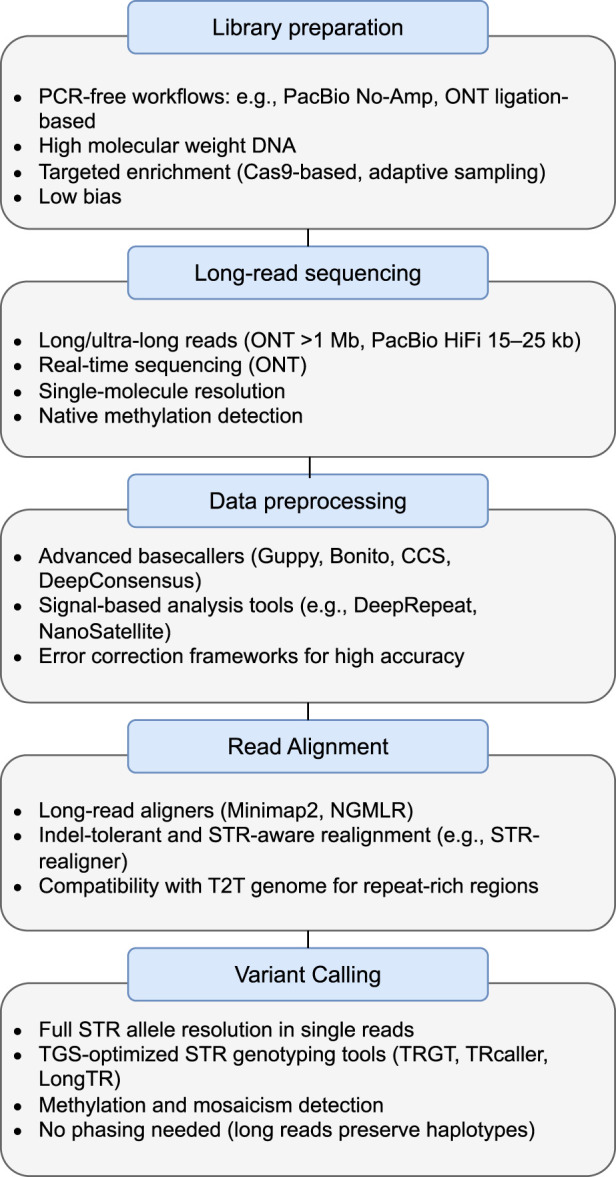
Overview of the STR analysis pipeline using third-generation sequencing (TGS) technologies. Each step—ranging from library preparation to variant calling—incorporates TGS-specific features such as PCR-free workflows, ultra-long reads, signal-based basecalling, indel-tolerant alignment, and STR-specific variant callers.

This section provides an in-depth journey through the intricacies of genetic investigation, from the initial processing of DNA to the extraction of meaningful genetic information. We explore the vital stages of library preparation, sequencing, data preprocessing, mapping, and variant calling, highlighting the fundamental principles and methodologies that underpin this dynamic field. Moreover, we describe the challenges and current state-of-the-art in each step. Figure 2 illustrates the complete pipeline for detecting STR variation using long-read sequencing technologies, highlighting TGS-specific features across each step—from library preparation to variant calling. The first step, library preparation, converts the genomic DNA sample (or cDNA sample) into a library of fragments which can then be sequenced on a TGS instrument. The sequencing step of the pipeline refers to the general laboratory technique for determining the exact sequence of nucleotides, or bases, in a DNA molecule. It tells scientists the kind of genetic information that is carried in a particular DNA segment. The next step, which is DNA preprocessing, particularly converts the “raw” signal data from the sequencing process into nucleotide sequences (A,C,T,G). The sequence alignment step is very crucial because it arranges the DNA (or protein) sequences to the reference genome to identify regions of similarity that may be a consequence of evolutionary relationships between the sequences. The last stage is variant calling, which identifies variants from the sequenced data. At every stage, from library preparation to variant calling, we maintain a flexible approach. This means that the pipeline can be adjusted, refined, or customized to better suit the characteristics of the samples, accommodate changes in project scope, or leverage improvements in sequencing technologies.

#### 3.1.1 Library preparation

As mentioned earlier in this section, the first stage of the pipeline is library preparation, which is a fundamental step in the process of sequencing long reads enriched with STRs. This journey begins with the isolation of high-quality genomic DNA from the biological sample of interest. This DNA serves as the raw material for subsequent analysis. Depending on the sequencing technology and specific objectives, genomic DNA can undergo controlled fragmentation to achieve the desired read length. However, some long-read technologies, such as Pacific Biosciences (PacBio) and Oxford Nanopore Technologies (ONT), can accommodate long input DNA molecules, obviating the need for extensive fragmentation. The high variability and repetitive nature of the STR regions require careful handling to preserve the integrity of these regions. Fragmented DNA molecules are subjected to end repair, resulting in blunt-ended fragments. Subsequently, sequencing adapters are ligated to the ends of these fragments. These adapters are essential for binding the DNA to the sequencing platform. In certain cases, size selection may be employed to enrich DNA fragments of a specific size range. This step is critical to ensure that the library contains fragments of the appropriate length for the sequencing platform in use.

##### 3.1.1.1 Challenges in library preparation

Library preparation for sequencing long reads enriched with STRs faces significant challenges in the context of technologies like PacBio and ONT. Maintaining high-molecular weight DNA is crucial, as excessive fragmentation can disrupt STR regions, leading to incomplete or biased sequencing data. Achieving an accurate representation of the full spectrum of STR lengths is difficult due to variability in repeat lengths; biases can occur during library preparation steps such as size selection and amplification. PCR amplification, while useful for increasing DNA quantity, introduces significant challenges when amplifying STR regions. PCR can result in artifact reads due to slippage during replication and has difficulties faithfully amplifying DNA sequences with extremely low complexity, such as STRs Trede et al. (2021). These limitations can lead to inaccuracies in STR length determination Jeanjean et al. (2025) and genotyping.

##### 3.1.1.2 State of the art in library preparation

Recent innovations have significantly improved the accuracy and efficiency of library preparation for long-read sequencing with STR enrichment. Amplification-free library preparation enabled by long-read sequencing technologies such as PacBio and ONT is particularly advantageous for sequencing STRs. By avoiding PCR amplification, these methods eliminate artifacts such as spurious deletions and PCR biases, leading to a more accurate representation of STR lengths Tsai et al. (2017); Gilpatrick et al. (2020). Amplification-free approaches are especially beneficial for the genome-wide observation of STRs. Advancements in targeted enrichment without amplification, such as PacBio’s No-Amp targeted sequencing and ONT’s adaptive sampling techniques, allow for the selective sequencing of specific genomic regions without PCR. These methods enhance the ability to observe STRs across the genome accurately, reducing the introduction of amplification-related errors Payne et al. (2021). Furthermore, optimized DNA extraction techniques that gently extract high-molecular-weight DNA help maintain the integrity of long STR regions Amarasinghe et al. (2020), and advanced enzymatic treatments have improved the efficiency and consistency of adapter attachment during end repair and ligation steps. Furthermore, computational correction of artifacts, such as the method developed by Raz et al. (2019), which calibrates a Markov model to predict and correct stutter patterns during amplification, enhances the accuracy of STR genotyping even when PCR cannot be completely eliminated.

#### 3.1.2 Long-read sequencing

The next step is sequencing, which serves as the central component of the pipeline, where the library is processed to generate long-read data. The prepared libraries are loaded onto the chosen sequencing platform, such as a PacBio Sequel instrument Loit et al. (2019) or an Oxford Nanopore device (MinION, GridION, or PromethION) Jain et al. (2015). These platforms rely on very distinct principles and exhibit a long-tailed distribution of kilobase reads; more than 1,000,000 bp reads have been captured with ONT (ultra-long protocol), which has no technical upper limit Payne et al. (2019). Compared to short-read platforms, the nucleotide error rate per-read (12%–15%) is significantly greater Rang et al. (2018). These technologies can identify epigenetic nucleotide variations directly since they do not require amplification of input DNA Pollard et al. (2018).

The sequencing process commences, involving the generation of sequence data in real-time. Long-read sequencing platforms are designed to produce extended sequences, often spanning thousands to tens of thousands of DNA bases Amarasinghe et al. (2020), Logsdon et al. (2020). These long reads encompass not only the STRs of interest, but also the surrounding genomic context. After the reads are generated, stringent quality control measures are implemented to identify and eliminate low-quality reads that may compromise the accuracy of subsequent analyses. There are several reasons why it is useful to sequence and understand the genomic location and expansion of the different STRs, the most relevant ones being (i) a better understanding of their biological function, (ii) the effect they have in STR expansion disorders and how this affects the prognosis for the patient, and (iii) the evolutionary effect they may have in the different biological functions, not only for humans but also for wild population conservations. Currently, the detection of repeat expansions for diagnosis is done using polymerase chain reaction (PCR) based fragment length analysis (PCR-FLA) or Southern blot assays Tankard et al. (2018), Bahlo et al. (2018), Chintalaphani et al. (2021). Recent studies utilizing long-read sequencing have reported an increasing number of human diseases associated with STR expansions Deng et al. (2020); Sone et al. (2019).

Long-read sequencing is a type of nucleic acid sequencing that produces genomic data by generating individual reads that are each derived from a single molecule that is thousands of nucleotides or more in length. Compared to short-read sequencing technologies, process modifications include minimal library preparation processes and real-time targeting of unfragmented DNA molecules, where the only limit is the generation of high molecular weight DNA for these purposes. While these technologies offer advantages in resolving repetitive regions, structural variants, and phasing, they are complementary to short read sequencing technologies, each having distinct strengths depending on the application Jeanjean et al. (2025). In recent years, long-read sequencing has gained increasing attention for its ability to accurately characterize short tandem repeats (STRs), particularly in complex or disease-associated loci.

Pacific Biosciences (PacBio) is a sequencing technology, commonly referred to as third-generation sequencing technology (TGS), that does not require a polymerase chain reaction (PCR) prior to sequencing Cao et al. (2015). It provides a single-molecule real-time (SMRT) sequencing platform Eid et al. (2009), Uemura et al. (2010) that employs circular consensus sequencing (CCS) to generate highly accurate (99.9%) high-fidelity (PacBio Hi-Fi) reads (see Supplementary Figure S1 in the Supplementary Material) that are between 15kb and 20 kb long Hu et al. (2021). Hi-Fi reads can be used across a wide range of SMRT sequencing applications, from whole genome sequencing for *de novo* assembly, comprehensive variant detection, epigenetic characterization, RNA sequencing, and more. The SMRT technique uses miniaturized wells, known as zero-mode waveguides, in which a single polymerase incorporates labeled nucleotides and light emission is measured in real-time.

SMRT sequencing has several advantages, notably its ability to produce long reads in a single read, spanning large structural variants and challenging repetitive regions that confound short-read sequencers. Another advantage is low GC bias, which allows PacBio systems to sequence through extreme-GC and AT regions that cannot be amplified during cluster generation on short-read platforms. Additionally, SMRT sequencing can detect DNA methylations while sequencing, since no amplification is performed on the instrument. Furthermore, when the human HG002/NA24385 genome was sequenced, this approach achieved a precision rate of 99.91% for single nucleotide variations (SNVs), insertions and deletions (95.98%), and structural variants (95.99%) Wenger et al. (2019). It is the first long-read sequencing technology widely deployed, well-suited for resolving complex genomic regions containing STRs. PacBio sequencers provide two types of reads: continuous long reads with high error rates (12%) and shorter circular consensus sequencing (CCS) reads with lower error rates (2%). One disadvantage of PacBio sequencing technology is its relatively high cost per base, which can be a limiting factor for some projects. Additionally, the high error level (14%) poses a challenge. To address this, hybrid sequencing approaches, combining short-read and PacBio methods, have been used Berbers et al. (2020). Moreover, the sequencing run time could be up to 20h, and the sequencing equipment is expensive (approximately 525k USD), which could be cost-prohibitive for smaller laboratories Hu et al. (2021).

Recent advancements in long-read sequencing technologies have introduced new instruments that enhance throughput and accuracy. PacBio’s Revio system with SPRQ Pacific Biosciences (2022), launched in late 2022, delivers up to 480 Gb of HiFi reads per day with 99.95% accuracy, utilizing high-density SMRT Cells and onboard deep learning for real-time basecalling. It also enhances epigenetic profiling by enabling direct detection of 5 mC and 6 mA modifications. Additionally, PacBio’s Vega system Pacific Biosciences (2024), announced in 2024, offers a benchtop long-read sequencer designed to make HiFi sequencing more accessible and affordable without compromising on data quality. Built on the same technology as Revio, Vega delivers HiFi reads with > 99.9% accuracy and supports read lengths up to 20 kb. The system also features on-instrument DeepConsensus, barcode demultiplexing, and compact BAM file outputs, enabling cost-efficient sequencing and analysis from a single device.

Another TGS technology that can generate long reads, which can be valuable for sequencing DNA data with short tandem repeats (STRs), is Oxford Nanopore Technologies (ONT). Unlike other strategies, ONT does not use polymerase at any stage of library preparation or sequencing, simplifying the process and eliminating the need for fluorescence detection. This unique sequencing approach utilizes a nanopore as a biosensor to sequence long DNA molecules Romagnoli et al. (2023). The principle involves the direct detection of nucleotide strands translocating through a protein pore embedded in a membrane, resulting in distinctive alterations in ionic current (see Supplementary Figure S2 in the Supplementary Material). It is a commercial nanopore-based high-throughput Shafin et al. (2020) long-read sequencing platform that can generate 1 Mb + long reads Miga et al. (2020).

The pore chemistry of this technology allows for the unbroken traversal of long sequences, with the production of high molecular weight DNA being the limiting factor, distinguishing standard long reads (10–100 kb) from ultra-long reads (above 100 kb) Miga et al. (2020), Jain et al. (2016), Shafin et al. (2020). Both long and ultra-long reads are stated to have an accuracy of 87%–98%, with raw reads correctly calling 91% and 93% of homopolymers at least five bases long Logsdon et al. (2020). The ONT read accuracy of 92%–93% limits this method to single nucleotide variant calling Jain et al. (2016). When it comes to the accuracy of ONT raw reads, it depends on the base-calling (translation of the electrical signal to DNA sequence) algorithm that is used, which continues to improve over time Rang et al. (2018). Nanopore long-reads can confidently map to repetitive regions of the genome, including centromeric satellites, acrocentric short arms, and segmental duplications Hu et al. (2021). Sequencing data is generated in real-time, enabling rapid data analysis and real-time monitoring of experiments. Compared to PacBio or second-generation sequencing technologies, ONT instruments offer advantages in cost, portability, and size, making them highly beneficial in low-income settings or field applications Quick et al. (2016). The main drawback of ONT as a long-read sequencing technology is the relatively high error rate (ranging from 2% to 15%), particularly in homopolymeric regions, compared to other sequencing technologies, which can be challenging for accurate STR analysis. However, as base-calling models improve, these high-error rates diminish over time McCombie et al. (2019). Furthermore, ONT errors are mostly systematic, making them more difficult to fix than random errors from greater coverage McCombie et al. (2019). Another disadvantage could be the base-calling complexity because it can be complex and computationally intensive.

Recent instruments developed by Oxford Nanopore Technologies (ONT) support a range of throughput needs for long-read sequencing applications such as STR genotyping. The PromethION 2 is a benchtop nanopore device with powerful GPU, running two high-output PromethION flow cells and could generate hundreds of gigabases Oxford Nanopore Technologies (2025). For higher-throughput requirements, the PromethION 24 and PromethION 48 platforms offer support for up to 24 or 48 independent flow cells, respectively, enabling the sequencing of thousands of human genomes annually Oxford Nanopore Technologies (2025). These devices are ideal for population-scale projects, offering real-time analysis, modular run flexibility, and high output per flow cell. Together, the PromethION family enables scalable STR genotyping across diverse study designs and sample sizes.

##### 3.1.2.1 Challenges of long-read sequencing of STRs

While Nanopore sequencing suffers from both random and systematic indel errors Menegon et al. (2017); Krishnakumar et al. (2018), which can make read alignment and SV detection more challenging, PacBio sequencing has a high rate of random false insertions Carneiro et al. (2012), which can be partially addressed by circular consensus sequencing to generate high-fidelity (Hi-Fi) reads Wenger et al. (2019) (although different strategies, such as linear consensus Li et al. (2016) or unique molecular identifiers Karst et al. (2021), can be used in order to reduce errors).

Targeted sequencing is a sequencing strategy where specific genomic regions are selected before sequencing. This is a cost-effective way of sequencing only the desired region, and not the whole genome leading to a higher output of the target sequence and easier analysis of the data. The majority of targeted sequencing library preparation approaches rely on PCR-based amplification, where the desired genomic region is amplified. As mentioned before, PCR has some limitations that can affect the sequencing of STRs. Some targeted enrichment approaches have been optimized for long-read sequencing, such as using a long-range PCR where the full fragment is amplified. Even if the full fragment is amplified, it still requires a PCR step that might lead to polymerase slippage or amplifying errors. The polymerase sllippage can artificially extend or contract the length of the repetitive element. For example, if a locus should consist of 12 adenines, during the sequencing process reads may be generated with just 11 or even 13. It also leads to the loss of epigenetic modifications, as the epigenetic mark will only be present in the original strand. The signal will be lost when sequencing Mastrorosa et al. (2023).

To avoid this, a couple of amplification-free enrichment methods have been developed. The no-amplification targeted sequencing method (no-amp) used by PacBio is based on CRISPR/Cas9 technology. A guide RNA (gRNA) will recognize the target DNA sequence and Cas9 cleaves it. The cleaved fragment will then be attached to adaptors that are used as a handle for capture using magnetic beads. The enriched region with the adaptors is then used for sequencing Mitsuhashi and Matsumoto (2020), Gilpatrick et al. (2020), Tsai et al. (2017). The same approach has also been used for ONT sequencing, where the adaptors that are added after the cleavage are specific for ONT sequencing López-Girona et al. (2020), Goldsmith et al. (2021).

Moreover, ONT has developed adaptive sampling which is a software-controlled enrichment. In this approach, the first few hundred base pairs of the molecule are sequenced and the program makes a decision if the molecule is “on target”. The user has to upload a document specifying the “on target” sequence or sequences. If the molecule is “on target” it will be sequenced, if it is “off-target” it will be ejected from the pore by reversing the current Martin et al. (2022).

High error rates: As already stated, long-read sequencing has a lower accuracy rate when compared to short-read sequencing, which makes it difficult for accurate STR sequencing. Nevertheless, PacBio and ONT are constantly improving their technologies. With the CCS approach PacBio has reduced their error rate from 12% to 2% Cao et al. (2015). The PacBio SMRT for Hi-Fi reads has an average read length of 20 kb with 99.9% accuracy. ONT is constantly improving the flow cells, nanopores, and motor proteins by looking for new proteins that are more accurate. To date, nine different versions of the system have been released: R6 (June 2014, R7 (July 2014), R7.3 (October 2014), R9 (May 2016), R9.4 (October 2016), R9.5 (May 2017), R10 (March 2019), R10.3 (January 2020), R10.4 (July 2022) (Wang et al., 2021). The R10.4 flowcell has an average read length of 100 kb for ultra-long reads with a 99% accuracy Marx (2023).

##### 3.1.2.2 State of the art for long-read sequencing

The advent of long-read sequencing technologies, such as PacBio and Oxford Nanopore, has revolutionized the study of STRs. These platforms are capable of producing longer reads, which is particularly advantageous for capturing the full span of STRs, including longer repeat regions. Long-read technologies have addressed challenges associated with variable STR lengths. The ability to sequence longer fragments helps in better resolving complex repeat structures, reducing ambiguities in interpretation and contributes to more accurate characterization of STRs.

Oxford Nanopore is the only sequencing technology which enables sequencing and insights in real-time, which allows researchers and scientists to monitor and analyze sequencing data as it is generated Wang et al. (2021). Real-time sequencing facilitates quicker identification of STRs, streamlining the workflow and enabling rapid insights into the repetitive regions of the genome. Meanwhile, PacBio’s Single Molecule Real-Time (SMRT) sequencing provides high-fidelity, long reads, addressing challenges associated with short-read technologies in accurately characterizing STRs. Both platforms offer improved base calling and error correction techniques, contributing to the enhanced accuracy of sequencing data. Additionally, PacBio’s Iso-Seq method is valuable for transcriptome analysis of STRs. Both technologies have seen efforts to reduce sequencing costs, making large-scale genomic studies involving STR analysis more accessible. Staying informed about the latest developments in these technologies is crucial for understanding their current state of the art.

#### 3.1.3 Data preprocessing

Third phase of the pipeline, DNA preprocessing, plays a pivotal role in transforming the primary raw signal data generated during long read sequencing into interpretable nucleotide sequences. This critical step involves basecalling, a process essential for translating raw signals into the corresponding nucleotide sequences Zhang Y.-Z. et al. (2020). Additionally, the DNA preprocessing stage may encompass error correction procedures, particularly when dealing with platforms known for higher error rates, such as Oxford Nanopore Delahaye and Nicolas (2021). Error correction is crucial to enhance the overall accuracy of the data, ensuring reliable identification and interpretation of Short Tandem Repeats (STRs) within the genomic sequences. However, long-read sequencing platforms have their own basecalling softwares or algorithms. For an example, basecallers used for Oxford Nanopore sequencing data are: Guppy, Albacore and SACall based on neural networks. Also, there are development versions of Guppy basecaller^1^, such as: Flappie, Scrappie, Taiyaki, Runnie, and Bonito. For the majority of users, Guppy basecaller often offers the highest accuracy and most reliable performance Wick et al. (2019). Development basecallers are frequently used to test features, for example, homopolymer accuracy, variation identification, or base modification detection, although they are not always optimised for speed or overall accuracy. Compared to SMRT basecalling, nanopore basecalling is inherently more advanced and offers a wider range of possibilities. Out of the 26 basecalling-related tools that are discovered, 23 are connected to nanopore sequencing. The majority of SMRT basecallers are created internally, and they need chemical version-specific training. Currently, CCS is the basecalling workflow Boža et al. (2017). However, there are several independent basecallers with various network architectures, the most well-known of which being Chiron Teng et al. (2018). Also, an important development in basecalling technology that holds significant promise for improving short tandem repeat (STR) analysis is Google’s DeepConsensus tool^2^. DeepConsensus is a deep learning-based approach designed to enhance the accuracy of CCS reads generated by Pacific Biosciences (PacBio) Hi-Fi sequencing platforms Baid et al. (2023). By employing neural networks to model and correct errors in the raw sequencing data, DeepConsensus produces high-accuracy consensus reads without additional sequencing passes.

##### 3.1.3.1 Challenges in data preprocessing

Despite the significance of DNA preprocessing in extracting meaningful information from raw signal data, several challenges are inherent in the context of Short Tandem Repeats (STRs) within long read sequencing. The variable lengths of STRs pose complexities in accurately assigning nucleotides during basecalling, demanding specialized algorithms capable of handling the intricacies of repetitive sequences. Moreover, error correction becomes a critical challenge, especially for platforms like Oxford Nanopore with higher error rates, as the correction process needs to discern genuine variations, such as STR expansions, from sequencing errors. Balancing the need for error correction without compromising the true variability of STR lengths is a delicate task, requiring careful consideration and development of advanced computational approaches tailored to the unique characteristics of STRs in long read sequencing data. Basecalling accuracy may be impacted by the context surrounding STRs, such as adjacent variants and flanking regions. Distinct genomic areas have distinct sequence contexts, which makes it difficult for basecallers to accurately determine STRs.

##### 3.1.3.2 State of the art for data preprocessing

Advanced basecalling techniques, exemplified by methods like those from Oxford Nanopore Technologies, have significantly improved the accuracy of sequence interpretation. The DNA preprocessing stage extends to error correction procedures, particularly vital for platforms with higher error rates such as Oxford Nanopore. Addressing challenges inherent in STRs, especially their variable lengths, requires specialized algorithms for accurate nucleotide assignment during basecalling. Furthermore, error correction is intricate, demanding algorithms capable of distinguishing genuine STR variations from sequencing errors. Striking a balance between robust error correction and preserving the true variability of STR lengths necessitates the development of sophisticated computational approaches tailored to the unique characteristics of STRs in long read sequencing data. The state-of-the-art in DNA preprocessing has made notable strides in overcoming these challenges, contributing to enhanced accuracy and reliability in the identification and interpretation of STRs within genomic sequences. The ongoing enhancement of the ONT base-calling algorithm consistently boosts read accuracy Wick et al. (2019), indicating the significance of repeating base calling for older data. ONT base-calling algorithm regularly improve the read accuracy, which suggests that repeating the base calling of older data is valuable. In contrast, the PacBio base-calling process is well-established, yielding BAM files with unaligned reads directly from the sequencing machine. Post-processing of subreads is imperative for Hi-Fi reads to condense consecutive sequenced DNA molecules into a high-quality consensus sequence. This post-processing occurs on the Sequel IIe system’s latest version, leading to a substantial reduction in overall data storage requirements.

#### 3.1.4 Read alignment

Once sequence reads are generated, the next step in the pipeline is alignment where reads are mapped to a reference genome sequence. The quality of sequence alignment is crucial, especially in the former approaches although usual alignment methods have difficulty in STR regions due to insertions and deletions caused by the variations of repeat numbers. To date, there are more than 80 read aligners that have been developed through the years Fonseca et al. (2012). The latest human reference genome assembly, released by the Genome Reference Consortium, was GRCh38 in 2017 Schneider et al. (2017). Several patches were added to update it, so the latest patch being GRCh38.p14 was published in March 2022^3^. However, a more complete reference, the T2T CHM13v2.0 assembly^4^ Chiu et al. (2024), which represents the first truly telomere-to-telomere human genome sequence, is now available on the UCSC Genome Browser as “hs1” and is anticipated to become the standard reference in the coming years. Furthermore, efforts are underway to develop a “human pan-genome” that captures genomic diversity from a wide range of global populations. Alignment software, often specialized for STR regions, accurately positions the reads within these repeat regions. This stage requires algorithms capable of handling repetitive elements adeptly.

The paper Fonseca et al. (2012) contains an exhaustive compilation of these read alignment tools. New sophisticated alignment methods that are indel (insertion or deletion) tolerant have been developed, enabling more accurate alignment of reads in STR loci. In this subsection we are going to present the alignment methods (tools) that are usually used for aligning long reads. Some commonly used alignment tools for long reads are: Minimap2 Li (2018), NGMLR (Next-Generation Mapping Long Read) Sedlazeck et al. (2018b), Blat Wang and Kong (2019), Bowtie2 (for Hybrid Mapping) Langdon (2015) and BWA-MEM (for Hybrid Mapping) Li (2013).

Minimap2 is a versatile long-read aligner that can efficiently align long reads to a reference genome. It is known for its speed and accuracy and is compatible with both PacBio and Oxford Nanopore data. On the other hand, NGMLR is specifically designed for aligning Oxford Nanopore long reads. It aims to improve the accuracy of alignments by considering the high error rates associated with Nanopore sequencing. It is notably good for aligning lengthy sequences and gapped mapping, which other rapid sequence mappers meant for short reads cannot do correctly. Blat is widely used sequence alignment tool. It is specifically good for aligning long sequences and gapped mapping, which other rapid sequence mappers meant for short reads cannot do correctly. While primarily is a short-read aligner, Bowtie2 can be used in hybrid mapping approaches. We can align short reads first using Bowtie2 and then use a long-read-specific aligner to refine the alignment of long reads in complex regions. Similar to Bowtie2, BWA-MEM is a widely used short-read aligner that can be used in hybrid mapping strategies in combination with long-read aligners. In the estimation of repeat numbers in a short tandem repeat (STR) region from high-throughput sequencing data, two types of strategies are mainly taken: a strategy based on counting repeat patterns included in sequence reads spanning the region and a strategy based on estimating the difference between the actual insert size and the insert size inferred from paired-end reads. Kojima et al. (2016) proposed a new dynamic programming-based realignment method for STR regions named STR-realigner. It takes sequence reads aligned with other methods and realigns sequence reads by dynamic programming manner with consideration of the corresponding STR repeat pattern as prior knowledge. By allowing the size change of repeat patterns with low penalty in STR regions they Kojima et al. (2016) expect an accurate realignment. Although a similar algorithm is adopted in a tool for detecting STR regions in PacBio reads based on a 3-stage modified Smith-Waterman, consecutive STR regions can be handled in the proposed algorithm, unlike the tool. In addition, clipping fragments, which are an essential feature for the realignment, are also considered in the proposed algorithm. By allowing insertions and deletions of repeat patterns in STR regions with repeatedly use of repeat units, accurate realignment of sequence reads is expected. The STR-realigner realigns query read R to a genome sequence, taking into account the multiple use of repeat patterns for prespecified STR regions.

##### 3.1.4.1 Challenges in read alignment

Aligning reads within genomic regions containing Short Tandem Repeats (STRs) poses distinct challenges in the context of long read sequencing technologies. The inherent variability and repetitive nature of STRs, compounded by the unpredictable length of repeats, complicate the accurate mapping of long reads to a reference genome. Traditional alignment methods, optimized for short reads, often struggle to precisely navigate through these complex regions, where the lengths of STRs can vary significantly between individuals. The presence of insertions and deletions within repetitive motifs further hinders alignment accuracy. Specialized tools for long read sequencing, such as Minimap2 and NGMLR, have been developed to address these challenges, yet the dynamic nature of STRs requires continual advancements in alignment algorithms. The need for indel-tolerant methods emphasizes the ongoing efforts to enhance the accuracy of read alignment, ensuring a comprehensive understanding of the complex genomic landscape enriched with STRs using long read sequencing technologies. Additionally, not all reads aligning to an STR locus are informative, and a trade-off exists between run time and tolerance to insertions/deletions (indels) in aligners like BWA. The need for gapped alignment in profiling STR variations linked to neurological diseases poses computational and processing time challenges.

##### 3.1.4.2 State of the art for read alignment

The current state of the art for read alignment of Short Tandem Repeats (STRs) using long-read sequencing technologies demonstrates substantial progress. Long-read sequencing technologies, such as Oxford Nanopore and Pacific Biosciences, offer extended reads that effectively span entire STR regions, minimizing challenges associated with repetitive sequences. Advanced algorithms tailored for STR detection and alignment, including graph-based approaches, enhance accuracy in variable-length STR regions. The integration of long-read data with short-read data in hybrid approaches and the ability to detect base modifications contribute to improved precision. Machine learning applications further improve alignment accuracy, while publicly available databases and benchmarking tools facilitate comprehensive evaluations. The field’s ongoing advancements underscore the continuous efforts to address challenges and refine methods for robust and accurate STR read alignment within long-read sequencing datasets.

#### 3.1.5 Variant calling

After the aligning (mapping) process of the reads, the last stage in the pipeline is calling variants from the alignment. The typical variant calling process includes sequencing, read mapping or *de novo* assembly, variant calling, filtering of false positives, and sometimes phasing. Fast and precise variant identification plays a crucial role in both research and clinical applications involving the sequencing of the human genome Goodwin et al. (2016). Any basic variant calling pipeline includes two key stages: read alignment against a reference genome sequence and variant calling itself. Hence, the quality of the reference genome sequence as well as properties of the software tools used for read alignment and variant calling all influence the final result. By examining the long-read sequencing data, variant calling of STRs utilizing long reads aims to identify variations in the repeat lengths of short tandem repeats within the genome. In repetitive regions, the same read can sometimes be aligned to multiple locations, further complicating the calculation of coverage (number of times a base is sequenced). Genotyping is the technology that detects small genetic differences that can lead to major changes in phenotype, including both physical differences that make us unique and pathological changes underlying the disease. Because there is no clear foundation for inferring homology between pairs of matched repeat units, genotyping microsatellite repeats from reference mapped reads is fundamentally different from calling SNPs or indels in non-repetitive sequence. Regardless of intervening alignment gaps, microsatellite genotypes must be allocated in terms of allele length or the number of sequenced bases inside a read separating the non-repetitive flanking boundaries linked to the reference. In addition, in order to securely establish an allele length, readings must span a complete repeat track. One of the genotyping tools used for STRs is TRcaller Wang et al. (2023). In the paper Wang et al. (2023) authors claim that this software program is one of the fastest and most accurate tandem repeat genotyping tool by far for both short and long Next-Generation Sequencing reads from Illumina, PacBio and Oxford Nanopore. Compared to popular software solutions, TRcaller^5^ claims that it could achieve higher accuracy (99% in 289 human individuals) in detecting TR alleles with magnitudes faster (e.g., 2 s for 300x human sequence data). The software takes as an input an aligned sequences in indexed BAM format (as well as with a BAI index file) and a target TR loci file in BED format. This tool outputs the TR allele length, allele sequences, and supported read counts in the sequence data. Another notable tool for tandem repeat genotyping is LongTR^6^, which is specifically designed for long-read sequencing data from platforms such as PacBio and Oxford Nanopore Ziaei Jam et al. (2024). This tool is an extension of HipSTR Willems et al. (2017) method, which was initially designed for genotyping Short Tandem Repeats (STRs) using Illumina sequencing data. LongTR utilizes a clustering strategy combined with partial order alignment and a hidden Markov model to accurately infer consensus haplotypes and score potential genotypes, particularly in complex and long repeats that challenge other methods. It supports multi-sample calling and incorporates technology-specific error models, making it highly suitable for comprehensive TR analysis. LongTR has demonstrated superior performance compared to other TR genotyping tools, especially in regions with complex structural variations, making it a valuable addition to variant calling pipelines focused on tandem repeats. Following the discussion of tools such as TRcaller and LongTR, another significant addition is TRGT (Tandem Repeat Genotyping Tool)^7^. TRGT provides a robust approach for analyzing and visualizing tandem repeats across the genome, specifically designed to work with PacBio Hi-Fi sequencing data Dolzhenko et al. (2024). Moreover, TRGT accurately determines the consensus sequences and methylation levels of specified TRs, supporting both repeat expansion detection and allele-specific visualization. With advanced visualization techniques, it helps researchers interpret complex tandem repeat variations, identifying methylation signals and mosaicism with finer repeat length resolution than existing methods. Therefore, TRGT serves as a valuable tool in large-scale genomic studies, enhancing variant calling pipelines by offering detailed insights into repetitive elements.

##### 3.1.5.1 Challenges in variant calling

Despite recent advances in sequencing technology, STR variations from long-read sequencing data pose remarkable challenges to variant detection methods compared to other mutation classes. In the context of long read sequencing, variant calling from Short Tandem Repeats (STRs) faces challenges, especially in repetitive regions, which could obstruct the precise and trustworthy analysis. One major challenge is the intrinsically greater error rates of long-read sequencing technology, which can cause false positive or false negative calls and make it more difficult to precisely detect differences in repeat length within STR loci. Furthermore, accurate variant detection is made more difficult by the complex repeat structures—interruptions and compound repeats, among others—that are unique to many STR loci. Ambiguities in the alignment of long reads to reference genomes, especially in repeated sections, impede the process even further and frequently lead to inaccurate and inaccurate alignments. Also, the stutter noise from PCR amplification during library preparation can create false repeat lengths, demanding explicit modeling and removal to enhance accuracy. Computational resources are also heavily strained by the computational demands of evaluating massive amounts of long-read sequencing data for STR variants. Despite significant progress, the accuracy and reliability of variant discovery from Next-Generation Sequencing data still have room for improvement.

##### 3.1.5.2 State of the art for variant calling

The cutting-edge landscape of variant calling for Short Tandem Repeats (STRs) through long-read sequencing technologies showcases a progression. Long-read sequencing platforms such as Oxford Nanopore and Pacific Biosciences have revolutionized variant calling by providing extensive read lengths, effectively spanning the intricate patterns of STR regions. Advanced algorithms, specifically tailored for repetitive sequences, have evolved to decipher the complexities of variable STR lengths, ensuring precise and accurate variant identification within long-read datasets. Employing graph-based approaches improves the accuracy of variant calling in regions rich in STRs, capturing nuanced relationships within repetitive sequences. The capability of long-read technologies, especially Oxford Nanopore, to detect base modifications significantly refines variant calling, distinguishing authentic STR variations from sequencing artifacts. Machine learning applications contribute to the refinement of variant calling strategies, leveraging computational intelligence to navigate the complexities of STR-rich genomic regions. Hybrid methodologies, fusing long-read and short-read data, represent a synergistic approach, leveraging the strengths of each to enhance overall precision in variant calling, particularly within challenging STR contexts. The integration of publicly available databases and benchmarking tools ensures rigorous evaluations and empowers researchers to select and optimize variant calling tools tailored for the unique features of STRs. This dynamic convergence of technological innovation, algorithmic sophistication, and integrative approaches reflects a robust and evolving state of the art in variant calling for STRs using long-read sequencing.

### 3.2 Computational STR-detection tools using long reads

Bioinformatic tools for short tandem repeat (STR) detection are very essential for efficiently processing and interpreting data from repetitive DNA sequences. Several useful tools - such as RepeatHMM Liu et al. (2017), Deep Repeat Fang et al. (2022), Straglr (Short-tandem repeat genotyping using long reads) Chiu et al. (2021), STRique Giesselmann et al. (2019), NanoRepeat Fang et al. (2023), NanoSTR Lang et al. (2023), NanoSatellite De Roeck et al. (2019), and WarpSTR Sitarčík et al. (2023) - have been developed to analyze and detect STR expansions in long-read sequenced data. These tools typically determine tandemly repeated motifs, such as dinucleotide (e.g., ACACAC) or trinucleotide (e.g., TATATATA) sequences, and estimate the number of repeat units. This repeat length information is crucial for understanding STR variability, which has implications in both clinical and population-level studies. The tools vary in their underlying algorithms, target applications (e.g., genotyping or methylation detection), supported sequencing technologies (e.g., PacBio or ONT), and accepted input formats such as FASTQ, BAM, or raw fast5 signal files. Choosing an appropriate tool often depends on the sequencing platform, input data type, and the desired analytical output.

Tools such as Straglr^8^ Chiu et al. (2021), is used for genome-wide scans for short tandem repeat (STR) expansions or targeted genotyping using long-read alignments. It was created to identify STR alleles using clustering and statistical modeling from long reads of at least 200 bp, so short reads were not intended for use with this tool. However, as input, it takes long read alignments sorted by genomic coordinates in BAM format against the reference genome. It suggests using Minimap2 aligner^9^. As an output, Straglr only reports a range of STR distributions rather than precise, correct STR allele sequences, which could not be sufficient for applications like forensics that demand precision allele calling.

DeepRepeat^10^ Fang et al. (2022) detects STRs from Nanopore electric signals that are in.fast5 format. They assume that the directly adjacent repeats share a similar signal distribution, so convert the ionic current signals into RGB channels and transform the problem into an image recognition problem (deep learning problem). It makes a prediction whether a given base in long reads is in repetitive region or not.

On the other hand, RepeatHMM^11^ Liu et al. (2020) takes long reads in.fastq from a subject as input and can also take a BAM file (aligned reads to the reference genome) as input to find more than 10 predefined trinucleotide repeats or a gene given by users, after all reads are well aligned to a reference genome. When RepeatHMM takes a set of reads as input, it uses a split-and-align strategy to improve alignments, performs error correction, and uses a hidden Markov model (HMM) and a peak calling algorithm based on the Gaussian mixture model to infer repeat counts. RepeatHMM allows users to specify error parameters of the sequencing experiments, thus automatically producing transition and emission matrices for HMM and allowing the analysis of both PacBio and Oxford Nanopore data. It’s prefined models are included for more than 10 well known trinucleotide repeats: AFF2, AR, ATN1, ATXN1, ATXN2, ATXN3, ATXN7, ATXN8OS, CACNA1A, DMPK, FMR1, FXN, HTT, PPP2R2B, TBP.

STRique^12^ Giesselmann et al. (2019) is a python package to analyze repeat expansion and methylation states of short tandem repeats (STR) in Oxford Nanopore Technology (ONT) long read sequencing data and HMM. In order to build a profile HMM, STRique uses flanking sequences and the repeating pattern, with match states corresponding to k-mers in these sequences. This model does not allow variations within the repetition. Following the raw signal’s alignment with the profile HMM, the copy number is determined by counting match states.

NanoRepeat^13^ Fang et al. (2023) detection tool performs a quantification of STRs from long-read sequencing data using Gaussian mixture models. On the other hand, NanoSTR^14^ Lang et al. (2023) is a software that uses nanopore sequencing data to determine target STRs. Compared with other analysis methods, this technique makes use of length-number-rank (LNR) data from reads and multisampling statistical analysis techniques to precisely genotype and correct STR markers. When it comes to data characteristics, NanoSTR successfully mitigates the unexpected insertions-deletions (indels) and non-random sequencing errors that come with nanopore sequencing Wang et al. (2021). As a result, it improves the effectiveness of sequencing data utilization, the rate at which STR genotypes are detected, and the precision with which STR profiling is performed. Additionally, NanoSTR has a good robustness, it is compatible with various sequencing platforms, and outperforms some analysis methods. However, NanoSTR faces several challenges and limitations. Firstly, the distribution, size, quantity, and sequencing depth of indels can significantly impact their performance, relying on LNR of reads for STR loci identification. Secondly, the method employs various threshold settings affecting typing performance, including rank difference and read number ratios. Thirdly, alignment software limitations may constrain the process. Fourthly, NanoSTR is designed for specific STR loci and isn’t suitable for genome-wide detection. Fifthly, sequencing data quality is crucial, influencing NanoSTR’s efficacy. Sixthly, the method’s parameters are based on sensitivity, specificity, and consistency assessments, allowing users to adjust settings accordingly. Further research is needed to evaluate NanoSTR’s performance with large sample sizes and validate its effectiveness with additional real-world data.

Furthermore, NanoSatellite^15^ De Roeck et al. (2019) is a novel algorithm that could effectively call GC-rich tandem repeats, expand alleles, and disrupt motifs by directly analyzing tandem repeats on raw PromethION squiggle data. It uses a dynamic time warping (DTW) algorithm that determines the most optimal alignment between two (unevenly spaced) time series. To determine the final copy number call, the results are clustered into two clusters. When it comes to accuracy, NanoSatellite outperforms Scrappie and Albacore, coming close to the accuracy of the Guppy “flip-flop”. While NanoSatellite’s relative standard deviation is lower than Guppy’s “flip-flop,” it is still a bit higher.

Another algorithm is WarpSTR^16^ Sitarčík et al. (2023), which is alignment-free and uses the raw signal from nanopore sequencing reads to determine the length of short tandem repeats (STR) in a genome. By modeling the STR locus with a finite-state automaton and adapting the dynamic time warping (DTW) algorithm Bellman and Kalaba (1959), the approach outperforms existing methods such as NanoSatellite and STRique. It efficiently locates the flanks and isolates the STR locus signal while addressing signal normalization issues and utilizing Bayesian Gaussian Mixture Models (GMMs) for genotype derivation. Evaluation against high-confidence variant calls demonstrates its superior accuracy compared to STRique, making it a promising advancement in genome analysis.

Table 1 emphasizes the bioinformatic tools that have been widely used in the detection of short tandem repeats (STRs) using long-read sequencing technologies. The tools are categorized according to input data type, suggested aligner (if applicable), and the nature of their output. This classification helps guide the selection of appropriate tools depending on the available data, analytical goals (e.g., genotyping, repeat quantification, methylation detection), and platform-specific considerations (e.g., ONT or PacBio compatibility).

**TABLE 1:** Summary of STR detection tools for long-read sequencing, categorized by input type, aligner, output, and notable features.

Tool	Input type	Suggested aligner	Output	Notes
STRique	FAST5, FASTQ	N/A (signal-based HMM)	STR repeat count, methylation state	ONT-specific; supports methylation profiling
DeepRepeat	FAST5	N/A	STR classification (per base)	Converts ionic signals into RGB for deep learning
NanoSatellite	FAST5	N/A	STR copy number via DTW clustering	Effective for GC-rich repeats; uses signal squiggles
WarpSTR	FAST5	N/A	STR length estimation	Alignment-free; DTW + GMM-based genotyping
Straglr	BAM	Minimap2	STR allele length distribution	Genome-wide STR scan or targeted genotyping; not for precise allele calls
RepeatHMM	FASTQ or BAM	split-and-align strategy to improve alignment	STR genotypes, repeat counts	Predefined trinucleotide loci; PacBio and ONT compatible
NanoSTR	FASTQ	Minimap2	STR genotypes with error correction	Robust against indels and sequencing noise
NanoRepeat	FASTQ or BAM	N/A	Quantified STR counts	GMM-based genotyping; targeted STR detection

## 4 Conclusion

The study of STRs has gained significant importance due to their role in genetic diversity, human disease, and forensic applications. However, STR analysis presents unique challenges, particularly in accurately characterizing repeat expansions, resolving complex repeat structures, and mitigating sequencing errors. Traditional short-read sequencing technologies struggle with STR detection due to their inability to span long repetitive regions, resulting in high false discovery rates and limited sensitivity.

Advancements in TGS technologies have revolutionized STR genotyping by enabling long-read sequencing with improved resolution. Despite their benefits, these technologies introduce new challenges, such as high error rates, amplification biases, and the need for specialized bioinformatics tools. To address these issues, various computational tools have been developed to optimize STR detection, genotyping, and variant calling. The integration of targeted sequencing approaches, error correction algorithms, and hybrid methods combining long and short reads continues to improve the accuracy of STR analysis.

Future research should focus on refining machine learning-based variant calling, improving cost-effective targeted enrichment techniques, and integrating epigenetic modifications into STR analysis. The development of more comprehensive reference databases and benchmarking tools will also be critical in improving the reliability of STR genotyping.

## Footnotes

1
https://github.com/nanoporetech

2
https://github.com/google/deepconsensus

3
https://www.ncbi.nlm.nih.gov/datasets/genome/GCF_000001405.40/

4
https://www.ncbi.nlm.nih.gov/datasets/genome/GCF_009914755.1/

5
https://github.com/XuewenWangUGA/TRcaller/

6
https://github.com/gymrek-lab/longtr

7
https://github.com/PacificBiosciences/trgt?tab=readme-ov-file

8
https://github.com/bcgsc/straglr

9
https://github.com/lh3/minimap2

10
https://github.com/WGLab/DeepRepeat

11
https://github.com/WGLab/RepeatHMM/tree/master

12
https://github.com/giesselmann/STRique

13
https://github.com/WGLab/NanoRepeat

14
https://github.com/langjidong/NanoSTR

15
https://github.com/arnederoeck/NanoSatellite

16
https://github.com/fmfi-compbio/warpstr

## Abbreviations

STRs, Short tandem repeats; SNPs, Single nucleotide polymorphisms; DNA, Deoxyribonucleic acid; SNVs, Single-nucleotide variations; SV, Structural variants; NGS, Next-generation sequencing; TGS, Third-generation sequencing; ONT, Oxford Nanopore Technologies; PacBio, Pacific Biosciences; Hi-Fi, High-fidelity; PCR, Polymerase chain reaction; SMRT, Single-molecule real-time; SINE, Short interspersed nuclear elements; LINE, Long interspersed nuclear elements; RBPs, RNA binding proteins; TRD, Tandem repeat disorder.
