# Supplementary material for: Get ready for short tandem repeats analysis using long reads-the challenges and the state of the art
Source: Front Genet. 2025 Jul 2;16:1610026. doi: 10.3389/fgene.2025.1610026 (PMC12263367; doi:10.3389/fgene.2025.1610026)
Supplement: Supplementary file 1 [file DataSheet1.pdf]

Supplementary Material

1 SUPPLEMENTARY FIGURES

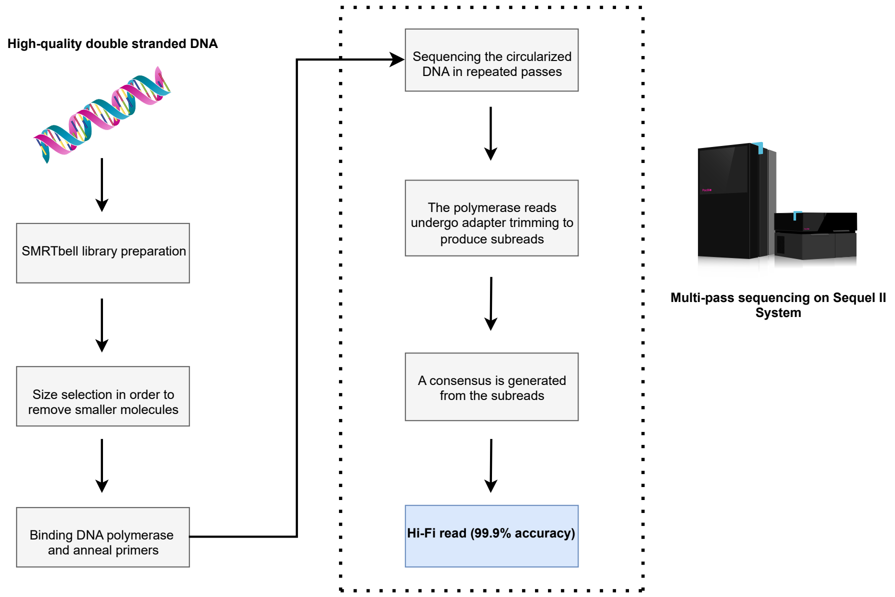

Figure S1. Schematic overview of PacBio HiFi sequencing. High-quality DNA is converted into SMRTbell libraries, followed by multi-pass sequencing and consensus generation to produce highly accurate HiFi reads (up to 99.9% accuracy).

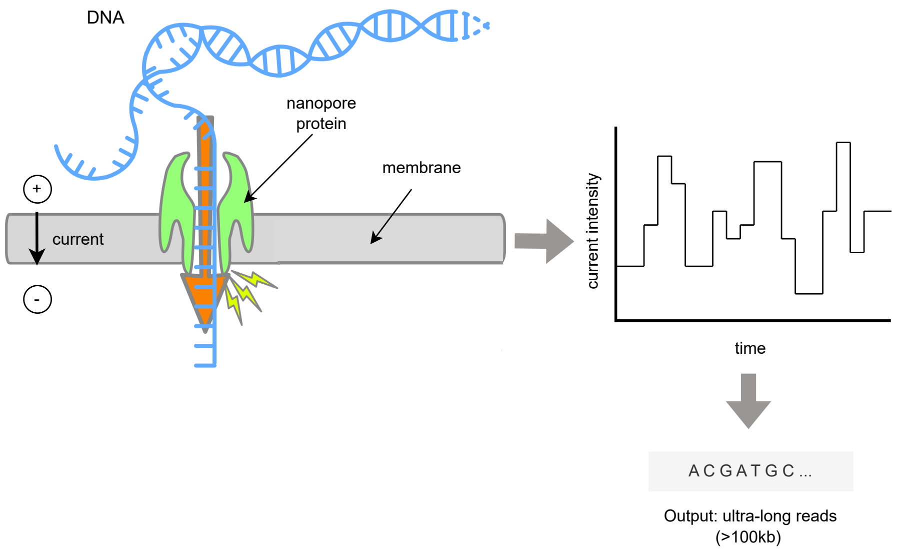

Figure S2. Schematic representation of Oxford Nanopore sequencing. Long DNA strands pass through a protein nanopore embedded in a membrane, disrupting the ionic current. These current changes are measured in real time and interpreted by basecalling software to generate ultra-long reads (>100 kb).
